# Venous Thromboembolism in Pediatric Musculoskeletal Infections: Diagnostic Challenges in a Resource‐Limited Setting

**DOI:** 10.1002/ccr3.72079

**Published:** 2026-02-17

**Authors:** Benedict Kusi Ampofo, Augustine Kwame Afful, Priscilla Konadu Sakyi, Justicia Amisah, Michael Bruce‐Smith, Dorcas Naa Dedei Aryeetey, Kwasi Adjepong Twum

**Affiliations:** ^1^ Directorate of Child Health Komfo Anokye Teaching Hospital Kumasi Ghana; ^2^ Directorate of Radiology Komfo Anokye Teaching Hospital Kumasi Ghana

**Keywords:** deep vein thrombosis, musculoskeletal infection, osteomyelitis, pediatric thrombosis, pulmonary embolism, resource‐limited settings, septic arthritis

## Abstract

Venous thromboembolism is a recognized but often underdiagnosed complication of pediatric musculoskeletal infections, particularly in low‐resource settings where access to Doppler ultrasonography, magnetic resonance imaging, and computed tomography pulmonary angiography (CTPA) remains limited. We describe two previously healthy children who developed deep vein thrombosis with pulmonary embolism secondary to acute musculoskeletal infection. Both presented initially with fever, severe limb pain, and rapidly progressive swelling, followed by the onset of tachypnoea and respiratory compromise. Doppler ultrasonography confirmed lower‐limb deep vein thrombosis in both cases, while computed tomography pulmonary angiography in one child demonstrated bilateral pulmonary emboli with pulmonary infarction. In the second child, pulmonary embolism was diagnosed based on clinical deterioration and characteristic chest radiographic findings in the absence of available computed tomography. Both children were managed successfully with intravenous antibiotics, therapeutic anticoagulation, and surgical drainage where indicated, although one required mechanical ventilation in the pediatric intensive care unit. These cases highlight the diagnostic challenges of venous thromboembolism complicating musculoskeletal infection in resource‐limited settings and emphasize the importance of maintaining a high index of suspicion when limb swelling worsens or respiratory symptoms develop. Bedside Doppler ultrasonography and timely referral remain essential tools for reducing morbidity when advanced imaging is inaccessible.

## Key Clinical Message

In children with musculoskeletal infection, disproportionate limb swelling or new‐onset respiratory compromise should prompt evaluation for venous thromboembolism. Bedside Doppler ultrasonography can support timely diagnosis and guide anticoagulation when advanced imaging is unavailable in resource‐limited settings.

## Graphical abstract

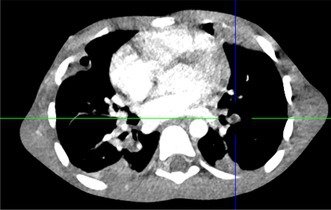

## 1 Introduction

Musculoskeletal infections (MSI) remain a common cause of pediatric hospital admissions across Sub‐Saharan Africa [1]. Although the association between MSI and venous thromboembolism (VTE) is well recognized in high‐income settings and carries a high fatality rate, cases are rarely reported from low‐resource regions [2]. This disparity likely reflects diagnostic constraints rather than true epidemiologic differences. Timely diagnosis of deep vein thrombosis (DVT) and pulmonary embolism (PE) relies heavily on Doppler ultrasonography and computed tomography pulmonary angiography (CTPA), modalities that are often unavailable, unaffordable, or difficult to access within many African health systems [3, 4].

Recent studies estimate the prevalence of VTE complicating pediatric MSI to be between 2% and 5%, substantially lower than earlier small studies that suggested higher rates [5, 6, 7, 8]. Despite this relatively low prevalence, VTE remains a clinically important complication, as delayed recognition may result in septic pulmonary embolization, respiratory failure, prolonged hospitalization and can be associated with significant morbidity and mortality [5, 9, 10, 11, 12]. In Sub‐Saharan Africa, where access to advanced imaging is limited, such complications are likely to be underdiagnosed.

We present two children with MSI complicated by DVT and PE managed in a resource‐limited setting. Rather than describing a novel association, these cases are presented to highlight system‐level diagnostic challenges, the reliance on incomplete or nonspecialized imaging, and the importance of heightened clinical suspicion when managing severe pediatric MSI.

## 2 Case Presentations

### 2.1 Case 1

A previously healthy 3‐year‐old boy developed a limp and progressive erythematous swelling of the left leg over 5 days, accompanied by severe pain and low‐grade fever. He was evaluated at a peripheral facility, where empirical intravenous ceftriaxone and azithromycin were initiated due to fever, localized erythematous leg swelling, severe pain with reduced weight‐bearing, and elevated inflammatory biomarkers, consistent with suspected acute bacterial MSI. Initial plain radiography demonstrated soft‐tissue swelling without cortical destruction or subperiosteal reaction. During this period, he developed a cough productive of blood‐stained sputum and worsening respiratory distress, prompting referral to our tertiary hospital.

On arrival, he was acutely ill with fever (40.1°C), tachycardia (171 bpm), tachypnoea (68 breaths per minute), and hypoxia (SpO^2^ 83% on room air). Chest examination revealed bronchial breath sounds and bilateral basal crepitations. The left leg was markedly swollen, warm, and erythematous with exquisite deep tenderness and pain on passive ankle dorsiflexion. The mid‐tibial circumference measured 24.5 cm compared with 19 cm on the contralateral side. Distal pulses and capillary refill were preserved.

Laboratory investigations showed anemia (Hb 8.9 g/dL), leucocytosis (22,390/μL with neutrophilic predominance), thrombocytopenia (132,000/μL), D‐dimer > 2000 ng/mL, and elevated ESR (70 mm/h). Blood cultures later isolated methicillin‐sensitive *Staphylococcus aureus* (MSSA).

**FIGURE 1:**
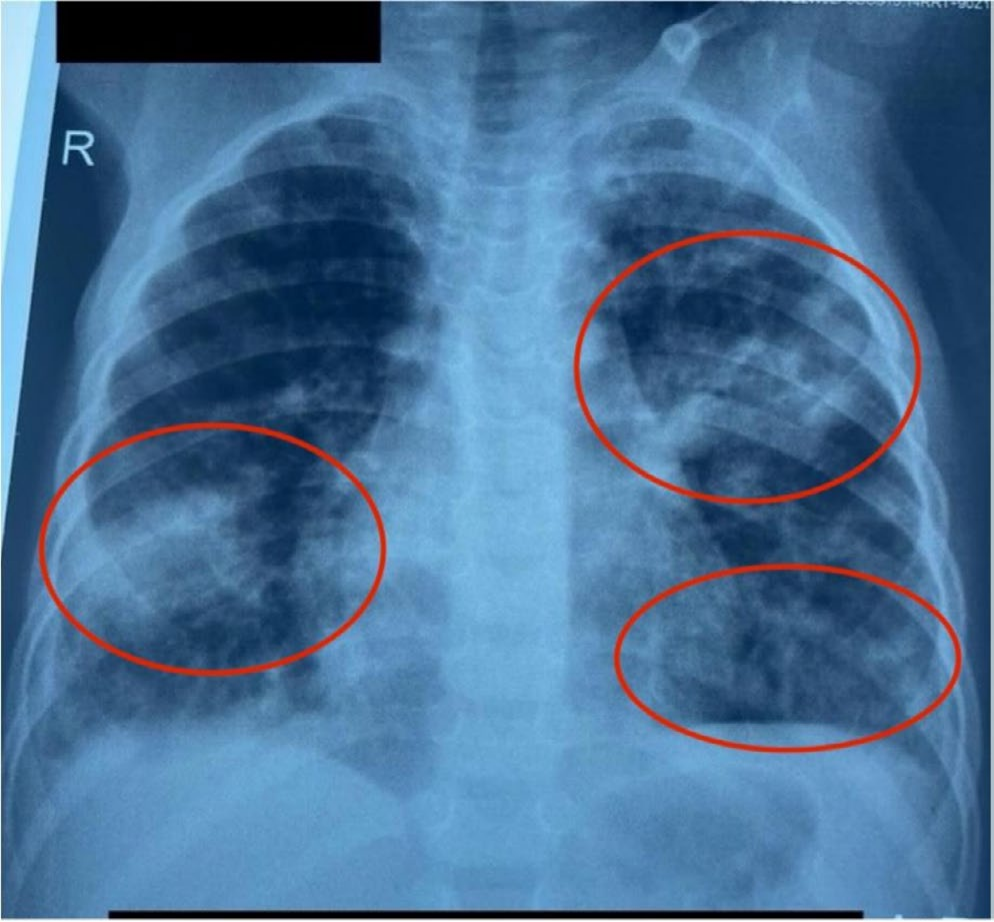
Frontal chest X‐ray showing diffuse poorly marginated nodular opacities and consolidation bilaterally (Red Ovals), suggestive of septic pulmonary emboli.

Doppler ultrasonography showed acute DVT involving the popliteal and posterior tibial veins, with periosteal elevation and subperiosteal fluid collection, and surrounding soft‐tissue oedema, features consistent with early tibial osteomyelitis, given the clinical context. While chest radiography demonstrated bilateral peripheral nodular opacities suggestive of septic emboli (Figure 1), CT pulmonary angiography and MRI could not be done due to diagnostic access limitations and cost.

In the context of acute respiratory deterioration, differential diagnoses included severe bacterial pneumonia, acute respiratory distress syndrome (ARDS), pulmonary hemorrhage, and septic pulmonary embolism (SPE). SPE was favored because the child had confirmed lower‐limb DVT, hemoptysis, sudden pleuritic respiratory decline, and chest radiographic findings of peripheral nodular and wedge‐shaped opacities rather than diffuse alveolar infiltrates typical of ARDS or lobar consolidation. Additionally, clinical improvement with combined intravenous antibiotics and anticoagulation further supported a diagnosis of SPE.

Surgical incision and drainage with tibial corticotomy yielded purulent material. He received intravenous ceftriaxone and clindamycin throughout his inpatient stay for treatment of acute osteomyelitis and was subsequently transitioned to oral clindamycin to complete a total antibiotic duration of 6 weeks. Therapeutic anticoagulation was initiated with enoxaparin at a dose of 1 mg/kg subcutaneously every 12 h and continued for a total duration of 12 weeks, consistent with current pediatric VTE management guidelines. Anti‐Xa monitoring was unavailable; therefore, dosing followed weight‐based pediatric protocols with close clinical surveillance for bleeding. Despite treatment, he developed worsening respiratory failure requiring pediatric intensive care and mechanical ventilation for 9 days. He improved steadily thereafter and was discharged home on anticoagulation after 31 days, remaining well at three‐month follow‐up. Key clinical events and interventions for this case are summarized chronologically in Table 1.

**TABLE 1:** Clinical timeline—case 1.

Day(s)	Key clinical events
Day 0	Onset of left leg pain, swelling, and fever
Days 1–4	Progressive swelling and difficulty bearing weight
Day 4	Evaluated at peripheral facility; empirical IV ceftriaxone + azithromycin initiated
Day 5	Developed hemoptysis and respiratory distress → referred to tertiary center; arrived hypoxic and tachypneic with markedly swollen, tender leg; labs consistent with sepsis; Doppler confirmed popliteal/posterior tibial DVT with features of tibial osteomyelitis; CXR showed peripheral nodular opacities; CTPA unavailable
Day 6	Tibial incision and drainage with corticotomy performed; IV ceftriaxone + clindamycin and therapeutic enoxaparin initiated
Days 6–15	Mechanical ventilation in PICU for 9 days due to respiratory failure
Day 31	Discharged on anticoagulation
3 months	Full recovery with no recurrence

### 2.2 Case 2

A previously healthy 6‐year‐old boy presented with a nine‐day history of fever, right hip pain, and limp. He was initially evaluated at a peripheral hospital, where empirical antibiotics were commenced due to persistent fever, focal right hip pain with limp, reduced weight‐bearing, and elevated inflammatory markers, consistent with suspected septic arthritis. During this period, he developed pleuritic chest pain, persistent cough, and progressive dyspnea requiring oxygen therapy, prompting referral to our center for further care.

On arrival, he was tachypneic (52 breaths per minute), tachycardic (144 bpm), and maintained oxygen saturation of 97% on 3 L/min of nasal oxygen. The right thigh was swollen (30 cm vs. 25 cm on the contralateral side), warm, and tender with significant pain on passive internal rotation of the hip, restricted range of motion of both the hip and knee, and refusal to bear weight. Distal pulses and perfusion were preserved. Chest examination revealed reduced breath sounds at the lung bases and pleuritic splinting on deep inspiration. Laboratory tests revealed leukocytosis (15,630/μL), markedly elevated CRP (196 mg/L) and ESR (46 mm/h), D‐dimer > 2000 ng/mL, and hemoglobin of 10.1 g/dL. Blood cultures isolated methicillin‐resistant *Staphylococcus aureus* (MRSA).

**FIGURE 2:**
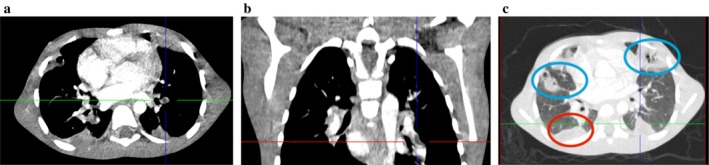
(a) Axial CTPA‐intraluminal filling defect, located centrally within the left lower lobe pulmonary artery (center of crosshair). (b) Coronal reformatted CTPA showing the left lower lobe pulmonary arterial intraluminal filling defect (center of crosshair). (c) Axial CT (lung window) showing wedge‐shaped peripheral consolidation in the left lower lobe (center of crosshair). Similar wedge‐shaped consolidation in the right middle lobe (Red Oval); with cavitating peripheral consolidations in the left upper lobe and right lower lobe (Blue Ovals), consistent with pulmonary infarcts.

Doppler ultrasonography showed a proximal femoral vein thrombosis contiguous with the inflamed hip joint, while hip ultrasonography demonstrated a significant joint effusion with echogenic purulent material, periarticular phlegmonous changes, and capsular distension consistent with septic arthritis; ultrasound‐guided aspiration of the hip joint yielded purulent fluid. CT pulmonary angiography demonstrated bilateral pulmonary emboli, including a central filling defect in the left lower lobe pulmonary artery, as well as cavitating consolidations and a wedge‐shaped peripheral opacity consistent with pulmonary infarction (Figure 2).

He underwent arthrotomy with drainage of the septic hip and received intravenous ciprofloxacin and clindamycin throughout his inpatient stay, followed by a transition to oral antibiotics to complete a total treatment duration of 6 weeks. Therapeutic anticoagulation was initiated with enoxaparin at a dose of 1 mg/kg subcutaneously every 12 h and continued for a total duration of 12 weeks. Anti‐Xa monitoring was not available; therefore, close clinical surveillance for bleeding was undertaken. His respiratory status gradually improved, oxygen support was discontinued after 11 days, and he completed a 30‐day inpatient course of antibiotics and anticoagulation. He remained well at the four‐month follow‐up. Key clinical events and interventions for this case are summarized chronologically in Table 2.

**TABLE 2:** Clinical timeline—case 2.

Day(s)	Key clinical events
Day 0	Onset of fever, right hip pain, and limp
Days 1–8	Persistent symptoms; managed peripherally for presumed septic arthritis
Day 9	Developed pleuritic chest pain, cough, and dyspnea → referred to tertiary center; arrived tachypneic requiring oxygen; right thigh swollen, warm, tender with reduced range of motion; labs showed inflammation; MRSA bacteremia; purulent hip aspirate
Day 10	Doppler confirmed proximal femoral DVT; CTPA demonstrated bilateral pulmonary emboli and pulmonary infarction; hip arthrotomy and drainage performed; IV ciprofloxacin + clindamycin and therapeutic enoxaparin initiated
Days 10–20	Gradual respiratory improvement; oxygen discontinued by Day 11
Day 30	Completed 30‐day inpatient IV antibiotic course
4 months	Full clinical recovery with no recurrence

## 3 Discussion

VTE complicating pediatric MSIs is uncommon but clinically significant, and its true incidence in Sub‐Saharan Africa is likely underestimated because diagnosis depends on imaging modalities that are not universally available. While recent larger cohorts from high‐income settings report thrombosis rates between 2% and 5% among children with osteomyelitis or septic arthritis [5, 6, 7, 8], the number of reports from African settings remains very small. This disparity almost certainly reflects differences in diagnostic capacity rather than fundamental epidemiological differences [2, 5, 7, 13]. The two cases described here illustrate not only the well‐known pathogenic relationship between infection and thrombosis, but also the practical diagnostic barriers encountered in a resource‐limited environment.

### 3.1 Anatomical Correlation Between Infection and Thrombosis

A key observation in both children is the anatomical proximity between the primary infection and the venous thrombosis. The thrombosis in *Case 1* involved veins draining the region of tibial osteomyelitis, and in *Case 2*, the thrombus was located in the proximal femoral vein adjacent to the infected hip joint. This spatial correlation aligns with current understanding that VTE in the context of MSI arises predominantly through localized septic thrombophlebitis rather than systemic hypercoagulable states [2, 5, 7]. Intense local inflammation results in endothelial injury, venous stasis is promoted by soft‐tissue swelling and limb immobility, and bacteremia may directly contribute to intravascular propagation of infection [2]. A similar infection–thrombosis–embolization sequence has been described in a recent pediatric case report by Najeeb et al., in which acute osteomyelitis was complicated by thrombophlebitis and SPE, underscoring how failure to recognize the musculoskeletal source may delay diagnosis and definitive management of VTE [14]. This pattern contrasts with malignancy‐related thrombosis or postoperative thrombosis, in which clot formation commonly occurs at venous sites remote from the primary pathology [2, 15]. The anatomical relationships observed in our cases, therefore, strongly support a causal link between the infection and thrombosis. This may also explain why inherited thrombophilias are frequently absent in such reported cases, as the pathophysiology is predominantly driven by localized inflammatory and bacterial factors rather than systemic coagulation abnormalities [6, 10, 16].

Neither child had a personal or family history of thrombotic disorders, and hemoglobin electrophoresis excluded haemoglobinopathies in both cases. Comprehensive thrombophilia screening was not performed due to resource constraints; however, the close temporal and anatomical relationship between infection and thrombosis strongly supports an infection‐driven mechanism consistent with previous reports [5, 7].

### 3.2 Diagnostic Challenges in Resource‐Limited Settings

These cases also highlight the diagnostic challenges that arise when advanced imaging modalities such as CTPA are not readily accessible. Both children initially presented to peripheral facilities lacking Doppler ultrasonography and radiology support, delaying recognition of thrombosis despite clinical signs that eventually proved significant. Even at the tertiary center, financial constraints prevented CTPA in one patient. In this context, diagnosis relied predominantly on carefully integrating clinical evolution, bedside ultrasonography, basic radiographic findings, and laboratory evidence of systemic inflammation. In *Case 1*, progressive respiratory compromise, bilateral nodular opacities on chest radiograph, and ultrasound‐confirmed DVT provided sufficient grounds to diagnose SPE despite the lack of confirmatory CTPA. This diagnostic pathway mirrors real‐world practice in many low‐resource settings and underscores the need for clinicians to interpret available evidence pragmatically rather than depend exclusively on ideal imaging [2].

### 3.3 Clinical and Biological Profile

Children who develop VTE as a complication of MSI may exhibit distinct clinical and biological profiles that could aid in early recognition [2, 5]. The mean age of our two patients was 4.5 years, and both were male. Studies by Bouchoucha and Hollmig reported higher mean ages of 8.1 and 10.9 years, respectively, though male predominance was consistent across series [5, 6].

Both our patients demonstrated markedly elevated inflammatory markers, with C‐reactive protein and erythrocyte sedimentation rate notably elevated at presentation. These findings align with observations by Bouchoucha et al. [5] and suggest the potential importance of such biological markers in monitoring treatment response and possibly developing risk assessment scores for predicting VTE in pediatric MSI patients, particularly in low‐resource settings where advanced imaging may be limited.

The development of SPE has often been associated with increased requirement for mechanical ventilation and intensive care admission [8, 10, 11, 12, 17], as was necessary in *Case 1*. Thus, this complication significantly prolongs hospitalization, with our mean length of stay being 30.5 days, consistent with reported means of 30.6 days and 33.5 days in similar cohorts [6, 9].

### 3.4 Clinical Implications and Practical Recommendations

Clinicians managing children with MSIs should maintain a high index of suspicion for VTE when limb swelling appears disproportionate to the local infection or when new respiratory symptoms emerge during the course of illness. Bedside Doppler ultrasonography—when available—is invaluable and often decisive [18]. In settings where formal radiology services are limited or unaffordable, point‐of‐care ultrasound can serve as the primary diagnostic tool guiding early anticoagulation [18]. Recognizing SPE on chest radiography remains useful, especially in facilities without access to CT imaging, and awareness of its radiographic manifestations should form part of routine pediatric emergency and infectious disease training.

Both methicillin‐susceptible and MRSA have been strongly associated with DVT in pediatric MSIs, a relationship thought to be mediated in part by virulence factors such as Panton–Valentine leukocidin [2, 6, 9, 10, 16]. Although molecular testing for PVL was unavailable in our setting, this association informed our decision to include clindamycin to provide coverage against potential PVL‐producing strains, given the severity of illness in both children [5, 19]. Therapeutic anticoagulation, surgical drainage, and intensive care support when needed remain cornerstones of management [2, 5, 6, 10, 16], with both our patients achieving complete recovery.

## 4 Limitations

Several limitations inherent to the resource‐constrained environment warrant acknowledgement. Archived ultrasound images were not retrievable, although findings were fully documented in patients' radiology reports. This challenge reflects broader infrastructural constraints rather than deficiencies in clinical assessment. Additionally, comprehensive thrombophilia screening and molecular characterization of the *Staphylococcus aureus* isolates were not performed because these investigations are not available within our institutional or regional diagnostic infrastructure, with financial constraints contributing secondarily. However, previous studies indicate that most children who develop thrombosis secondary to MSI do not have inherited prothrombotic conditions [2, 5, 10, 16]. The close temporal and anatomical alignment between infection and thrombosis and the favorable response to combined therapy strongly support our diagnoses and thus suggest an infection‐driven mechanism.

Taken together, these cases illustrate the importance of early recognition of VTE in children with MSIs, particularly in regions where access to advanced diagnostic imaging is limited. They underscore the practical value of point‐of‐care ultrasonography, the need for clinicians to integrate clinical and basic radiologic findings when definitive imaging is unavailable, and the necessity of strengthening diagnostic capacity across Sub‐Saharan Africa to reduce preventable morbidity in this vulnerable population.

## 5 Conclusion

VTE is an uncommon but serious complication of pediatric MSI. In resource‐limited settings, delayed diagnosis often results from restricted access to advanced imaging. Clinicians should maintain a high index of suspicion for DVT and PE when MSI is accompanied by progressive limb swelling or respiratory distress. Bedside Doppler ultrasonography, early anticoagulation, and timely referral are critical strategies for reducing morbidity where diagnostic resources are constrained.

## Author Contributions

**Benedict Kusi Ampofo:** conceptualization, writing – original draft, writing – review and editing. **Augustine Kwame Afful:** conceptualization, writing – original draft, writing – review and editing. **Priscilla Konadu Sakyi:** writing – original draft, writing – review and editing. **Justicia Amisah:** conceptualization, writing – original draft, writing – review and editing. **Michael Bruce‐Smith:** conceptualization, writing – original draft, writing – review and editing. **Dorcas Naa Dedei Aryeetey:** data curation, writing – original draft, writing – review and editing. **Kwasi Adjepong Twum:** conceptualization, supervision, writing – original draft, writing – review and editing.

## Funding

The authors have nothing to report.

## Ethics Statement

Ethical approval was waived for this case report in accordance with institutional policy for descriptive reports of anonymized clinical cases.

## Consent

Written informed consent was obtained from the legal guardians of both children for publication of the clinical details and accompanying images.

## Conflicts of Interest

The authors declare no conflicts of interest.

## Data Availability

The data that support the findings of this study are available from the corresponding author upon reasonable request.
